# The Potential Predictive Biomarkers for Advanced Hepatocellular Carcinoma Treated With Anti-Angiogenic Drugs in Combination With PD-1 Antibody

**DOI:** 10.3389/fimmu.2022.930096

**Published:** 2022-07-07

**Authors:** Chenxi Liu, Sihui Zhu, Yanbing Dong, Jie Shao, Baorui Liu, Jie Shen

**Affiliations:** ^1^ Comprehensive Cancer Centre of Drum Tower Hospital, Medical School of Nanjing University, Clinical Cancer Institute of Nanjing University, Nanjing, China; ^2^ Comprehensive Cancer Centre of Nanjing Drum Tower Hospital, Clinical College of Nanjing Medical University, Nanjing, China

**Keywords:** hepatocellular carcinoma, neoantigen reactive T cells, immunotherapy, PD-1 antibody, biomarker

## Abstract

**Background:**

Based on molecular biomarkers, anti-angiogenic drugs in combination with programmed cell death protein 1 (PD-1) antibodies can screen the potentially beneficial populations with hepatocellular carcinoma (HCC) and predict the efficacy after treatment. Therefore, we aimed to study predictive molecular biomarkers to improve the effectiveness of immuno-targeted combination therapy for HCC.

**Patients and Methods:**

Baseline clinical data, blood samples, and imaging data of the first evaluation after two cycles of treatment were collected for 40 patients with advanced HCC who underwent combination therapy, and then these data were compared according to the efficacy. Since 15 patients had complete hematology samples, we additionally tested the T lymphocyte subpopulations of these 15 patients and also compared them according to the efficacy. In addition, we also selected five patients who benefited the most from the combination therapy and five patients with the worst curative effect for gene detection based on survival time and efficacy evaluation. Finally, the relationship between certain clinical characteristics, laboratory indicators, specific T lymphocyte subpopulations, gene mutations and the response of immuno-targeted combination therapy for HCC was evaluated.

**Results:**

The high levels of CD3^+^CD4^+^CD279^+^, CD3^+^CD8^+^CD45RO^+^CD62L^+^T lymphocytes and tumor mutational burden (TMB) were associated with good efficacy of the combination therapy (P=0.03, P<0.01 and P=0.03). The high levels of CD3^+^CD4^+^CD28^+^ T lymphocytes were associated with poor efficacy of the combination therapy (P=0.02). The high mutation frequency of TP53 and ARID1A appeared in the non-response cohort. In addition, amplification mutation of 11q13-CCND1, FGF3, FGF4, and FGF19 was found in a patient with hyperprogression (HP).

**Conclusions:**

The certain clinical characteristics, laboratory indicators, specific T lymphocyte subpopulations, and gene mutations established in this paper were potential predictive biomarkers for HCC patients treated with combination therapy.

## Background

In China, hepatocellular carcinoma (HCC) ranks the top five most frequently diagnosed cancer types that have high morbidity and mortality (1). So far, immune checkpoint inhibitors (ICIs) against programmed cell death protein 1 (PD-1), cytotoxic T lymphocyte antigen-4 (CTLA-4), and so on have brought new hope for the treatment of advanced HCC, especially in combination with targeted drugs, which can significantly improve the long-term prognosis of potentially beneficial populations. The GO30140 research has shown that patients with advanced HCC can obtain a progression-free survival (PFS) of 7.3 months and overall survival (OS) of 17.1 months when using the first-line combination of atezolizumab and bevacizumab (2). Imbrave150 research has shown that the global objective response rate (ORR) of atezolizumab in combination with bevacizumab can reach 27.3% in the treatment of unresectable HCC (3). The median overall survival (mOS) reaches 19.2 months (17.0-23.7 months) (HR=0.66; 95% CI: 0.52-0.85) (4), and the mOS of the Chinese subgroup is 24 months (17.1-NE) (HR=0.53; 95% CI: 0.35-0.80) (5).

Although combination therapies have achieved significant effects on the treatment of HCC, the less than 27.3%-46% response rate of drugs and high treatment costs have greatly restricted the application of immuno-targeted combination therapy (3, 6). Therefore, it is urgently necessary to improve the efficiency of combination therapy and identify potentially beneficial populations through laboratory characteristics, hallmark T lymphocyte subsets and other features.

The Tasuku Honjo team, who won the 2018 Nobel Prize in Physiology or Medicine, has discovered PD-1 and programmed cell death ligand 1 (PD-L1) (7). The binding of PD-1 and PD-L1 plays an important role in the mechanism underlying the tumor immune escape (8, 9), suggesting that inhibiting the interaction can mediate the body’s anti-tumor activity. Since then, immunotherapy has been widely used in the field of tumor treatment.

The multi-kinase inhibitor sorafenib has become the standard treatment for HCC patients without indications for surgery since 2007 (10). However, its clinical efficacy is not satisfactory. The current emergence of anti-angiogenic drugs has broken this deadlock. A REFLECT study has reported the lenvatinib monotherapy for HCC patients, with a PFS of 7.4 months and an ORR of 24.1% (11), which is significantly higher compared with the sorafenib monotherapy (PFS of 3.7 months, ORR of 9.2%) (11).

As far as immunotherapy is concerned, the GO30140 study has shown that the ORR of atezolizumab as a single agent in advanced HCC is 17%, and the PFS is 3.4 months (1.9-5.2 months)(HR=0.55, 80% CI: 0.40-0.74 P=0.0108) (2). The Checkmate459 study has shown that when nivolumab monotherapy is used for unresectable HCC, the OS is 16.4 months (P= 0.075), PFS is 3.7 months (95%CI: 3.1-3.9), and ORR is 15% (12). The above-mentioned data all indicate that no more than 20% patients can benefit more from single PD-1 antibody treatment. A large number of research data in the past 5 years have shown that anti-angiogenic drugs in combination with PD-1 antibody therapy can further improve patient’s survival, such as Imbrave150 (3), Rescue (13), Orient-32 (14), Keynote-524 (6) and so on.

At present, clinical trials of different types of anti-angiogenic drugs and PD-1 antibodies in the treatment of HCC are actively carried out in various tumor centers. However, the relevant indicators that can predict the potentially beneficial populations of combination therapy are only reported in malignant melanoma, non-small cell lung cancer (NSCLC), and other tumor types. There is no definite evidence in the field of HCC treatment.

In the present study, we performed statistics on the clinical characteristics, laboratory indicators, multiple T lymphocyte subtypes, and gene mutations of patients with advanced HCC in clinical trials who underwent immuno-targeted combination therapy. Moreover, we aim to explore sensitive response predictors of HCC combination therapy, and analyze the relationship between predictors and the sensitivity of combination therapy.

## Patients and Methods

### Study Design and Participants

All specimens and relevant clinical data were obtained from the department of oncology, Drum Tower Hospital Affiliated to Medical School of Nanjing University. Baseline clinical data, blood samples, and imaging data of the first evaluation after two cycles of treatment were collected for 40 patients with advanced HCC treated with anti-angiogenic drugs in combination with PD-1 antibody. Patients started the combined treatment from May 2018. Since 15 patients had complete hematology samples, we additionally tested the T lymphocyte subpopulations. Based on the evaluation of response, the results of gene mutations of five patients each with the greatest and the worst clinical benefit were obtained. All HCC patients were confirmed by histopathology. Clinical data including the patient’s age, gender, histopathological diagnosis, tumor location and stage, lymph node metastasis, and evaluation data after combination treatment. Clinical characteristics of the patients were summarized in **Table 1**. Informed consent was obtained from all patients. The protocols for our study were approved by the Human Research Protective Committee of Drum Tower Hospital.

**Table 1:** Baseline Demographic and Clinical Characteristics of Patients Receiving Combination Therapeutics (N =40).

Characteristic	No. (%)
**Sex**	
Male	36 (90)
Female	9 (10)
**Age, years**	
Median	57.1
Range	40-73
**Age group, years**	
<65	31 (77.5)
≥65	9 (22.5)
**No.of involved disease site per patient**	
1-2	7 (17.5)
3-4	28 (70.0)
≥5	5 (12.5)
**No.of target lesions/total lesions**	
Median	0.58
Range	0.33-1
**Total diameter of target lesion,mm**	
Median	51.88
Range	11.04-150.29
**Involved disease sites**	
Liver	29 (72.5)
Lung	13 (32.5)
Lymph nodes	11 (27.5)
Bone	13 (32.5)

### Assessments

The blood sample was collected before the patients’ first treatment, focusing on the patients’ lymphocyte and neutrophil counts, and calculating the neutrophil to lymphocyte ratio (NLR). The first imaging evaluation was carried out within 28 days before the patients’ initial treatment and was repeated every two cycles after medication. The Independent Radiological Review Committee (IRRC) evaluated the response according to RECIST V1.1. Since 15 patients had complete hematological samples, their T lymphocyte subpopulations were additionally tested. The peripheral venous blood of 15 patients were collected before treatment. CD3-FITC, CD19-PE, CD16-PE CD279-PE, CD223-PE, CD366-PE, CD137-PE, CD62L-PE, CD27-PE, CD4-PerCP, CD8-PerCP, CD8-APC, CD56-APC, CD45RO-APC, CD28-APC, IgG1 k, IgG2ak were used for direct labeling and staining with multiple combinations. The T lymphocyte subpopulations were detected by flow cytometry (The data of fluorescence-activated cell sorting were list in the **Supplementary Materials**). The five patients who benefited the most from the combination therapy and the five patients with the worst curative effect were selected for gene detection. The samples of gene detection came from tumor tissue and hematological samples. Comprehensive genomic alteration analysis of the tumor and matched blood samples were performed with an assay panel that captured 450 cancer-related genes and selected introns of 38 genes frequently rearranged in cancer (YuansuTM, OrigiMed). The genomic profile was produced using the NGS-based YuanSuTM 450 gene panel. The genes were captured and sequenced with a mean coverage of 900× by using Illumina NextSeq 500.

### Statistical Analysis

**Figure 1:**
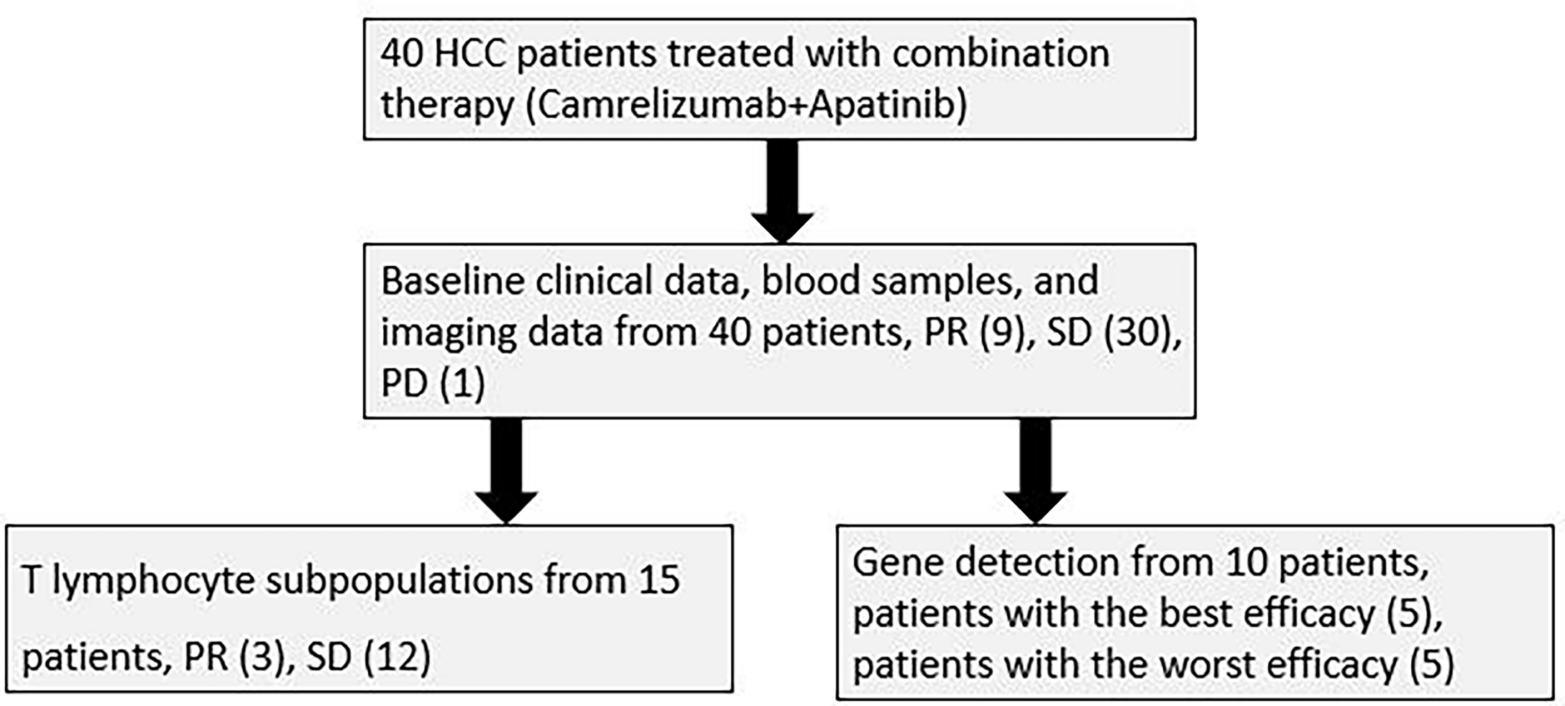
The overall work flow of the whole study.

As demonstrated by the overall workflow (**Figure 1**), pre-treatment clinical data, blood samples and imaging data were collected for 40 patients, and then these data were compared according to the efficacy. Among them, there were 9 partial remission (PR) patients named as Group PR, 30 stable disease (SD) patients and 1 progressive disease (PD) patient named as Group SD/PD at the first evaluation. T lymphocyte subtypes were measured in 15 patients, and the effect evaluation were also compared. Among them, there were 3 patients with PR and 12 patients with SD. Finally, we conducted gene detection on 10 patients based on the response evaluation. All statistical analyses were performed using SPSS 20.0. Chi-square test was used for qualitative data and t-test was used for quantitative data. P<0.05 was considered to be statistically significant. The Kaplan-Meier method was used to estimate PFS and OS.

## Results

Among 40 patients who received clinical data collection, hematological sample analysis, and imaging evaluation, 9 patients achieved PR, and 31 patients achieved SD/PD. The differences between the two cohorts were listed in **Table 2A**.

**Table 2A:** Differences in clinical data, hematology samples and imaging evaluations between the PR and SD/PD (n=40): PR (n=9), SD/PD (n=31).

	PR	SD/PD	t	P
gender	59.88 ± 10.43	56.29 ± 8.64	1.05	0.300
ANC	2.80 ± 1.03	3.95 ± 1.88	-1.76	0.087
LC	1.41 ± 0.59	1.53 ± 0.94	-0.36	0.724
NLR	2.48 ± 2.07	3.53 ± 2.70	-1.07	0.291
Target lesion/total lesion	13.26 ± 7.58	14.99 ± 8.28	-0.56	0.577
Liver metastasis	6 (66.67)	23 (74.19)	0.0004	0.983
Bone metastasis	0	1 (3.23)	/	1.00
Lung metastasis	4 (44.44)	9 (29.03)	0.216	0.642
Lymph node metastasis	3 (33.33)	8 (25.81)	0.0004	0.983

ANC, Absolute Neutrophil Count; LC, lymphocyte count.

**NLR** Although there was no significant statistical difference in NLR (2.48 ± 2.07 and 3.53 ± 2.70) (P=0.291), there was still a trend that the PR group was lower than the SD/PD group.

**Metastasis** Among lung metastases, the PR group accounted for 44.44%, and the SD/PD group accounted for 29.03%. Although there was no significant statistical difference, it seemed that patients with lung metastasis had a higher rate of PR.

**Figure 2:**
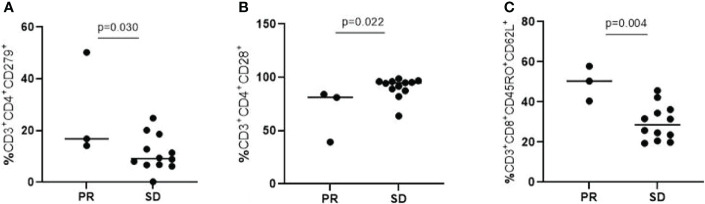
T lymphocytes with surface molecules expressing CD3+CD4+CD279+ **(A)**, CD3+CD4+CD28+ **(B)**, CD3+CD8+CD45RO+CD62L+ **(C)** showed significant statistical differences (P=0.030, P=0.022, P=0.004).

**Lymphocyte Subpopulations** Among 15 patients who received flow cytometry for the detection of lymphocyte subpopulations, 3 patients achieved PR, and 12 patients achieved SD. The differences between the two cohorts were listed in **Table 2B**. Among them, T lymphocytes with surface molecules expressing CD3^+^CD4^+^CD279^+,^CD3^+^CD4^+^CD28^+^, CD3^+^CD8^+^CD45RO^+^CD62L^+^ showed significant statistical differences (**Figure 2**; P=0.030, P=0.022, P=0.004). The high expression of CD3^+^CD4^+^CD279^+^ and CD3^+^CD8^+^CD45RO^+^CD62L^+^ T lymphocytes indicated a good prognosis for the patient. The high expression of CD3^+^CD4^+^CD28^+^ T lymphocytes suggested that immuno-targeted combination therapy was not effective.

**Table 2B:** Differences in lymphocyte subpopulation between the PR and SD (n=15): PR (n=3), SD (n=12).

	PR	SD	t	P
CD3^+^	71.06 ± 12.74	59.39 ± 12.96	-1.40	0.185
CD3^+^CD8^+^	42.37 ± 6.11	55.18 ± 11.00	1.91	0.079
CD3^+^CD4^+^	35.13 ± 15.89	33.01 ± 11.16	-0.27	0.788
CD8^+^/CD4^+^	150.0 ± 98.04	193.9 ± 95.61	0.71	0.491
CD56^+^CD16^+^	11.67 ± 3.48	20.09 ± 10.14	1.38	0.189
CD3^+^CD19^+^	6.63 ± 4.37	8.83 ± 7.69	0.47	0.648
CD3^+^CD8^+^CD279^+^	15.83 ± 14.52	13.79 ± 8.46	-0.33	0.748
CD3^+^CD8^+^CD223^+^	2.33 ± 3.78	0.717 ± 0.529	-1.60	0.133
CD3^+^CD8^+^CD366^+^	8.20 ± 9.68	5.51 ± 4.46	-0.74	0.470
CD3^+^CD8^+^CD137^+^	0.53 ± 0.75	0.67 ± 0.60	0.33	0.747
**CD3^+^CD4^+^CD279^+^**	**27.06 ± 20.07**	**11.18 ± 6.92**	**-2.43**	**0.030**
CD3^+^CD4^+^CD223^+^	0.63 ± 0.92	0.60 ± 0.39	-0.10	0.921
CD3^+^CD4^+^CD366^+^	2.70 ± 4.25	1.07 ± 0.81	-1.38	0.191
CD3^+^CD4^+^CD137^+^	0.73 ± 0.58	0.52 ± 0.52	-0.61	0.554
CD3^+^CD8^+^CD27^+^	72.76 ± 14.55	73.51 ± 14.04	0.08	0.935
CD3^+^CD8^+^CD28^+^	70.90 ± 21.23	72.67 ± 15.68	0.17	0.871
CD3^+^CD4^+^CD27^+^	60.83 ± 26.73	82.43 ± 12.73	2.13	0.053
**CD3^+^CD4^+^CD28^+^**	**68.20 ± 25.07**	**90.54 ± 9.66**	**2.61**	**0.022**
**CD3^+^CD8^+^CD45RO^+^CD62L^+^**	**49.56 ± 8.68**	**29.65 ± 8.79**	**-3.52**	**0.004**
CD3^+^CD4^+^CD45RO^+^CD62L^+^	44.46 ± 16.18	57.08 ± 10.17	1.73	0.107
CD3^+^CD8^+^CD45RO^+^CD62L-	34.06 ± 10.72	48.52 ± 11.51	1.96	0.071
CD3^+^CD4^+^CD45RO^+^CD62L-	24.00 ± 12.35	26.25 ± 6.67	0.45	0.663

Bold values means that there are statistical differences in these T lymphocyte subpopulations.

**Figure 3:**
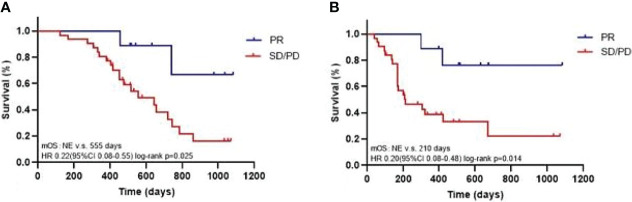
Kaplan-Meier analysis of OS **(A)** and PFS (mRECIST; **B**).

**Figure 4:**
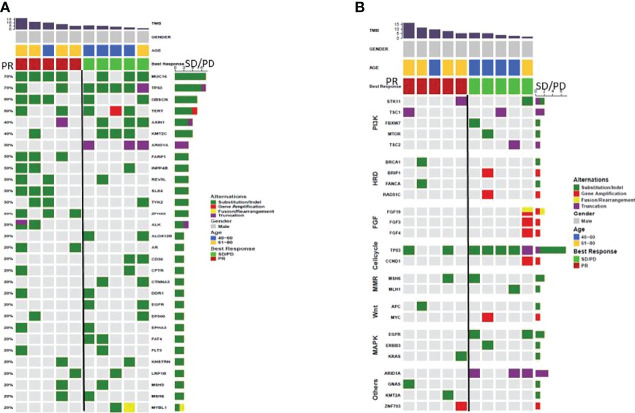
**(A)** difference in gene mutation rate between PR (n=5) and SD/PD (n=5), **(B)** difference in signaling pathway between PR (n=5) and SD/PD (n=5).

**Gene Mutations** The OS (**Figure 3A**, 95% CI 0.08-0.55; p=0.025) and PFS (**Figure 3B**, 95% CI 0.08-0.48; p=0.014) of the patients evaluated as PR (n=9) for the first imaging assessment based on RECIST V1.1 were significantly longer than those of SD/PD patients (n=31). This suggested that the first evaluation of the efficacy could predict the long-term prognosis. So we selected the most and the least clinical benefit five patients each for gene detection and compared them based on the efficacy. The difference in gene mutation frequency between the two cohorts was shown in **Figure 4A**, and the difference in signal pathways was shown in **Figure 4B**. Among them, ARID1A and TP53 had a higher proportion in the SD/PD cohort(60% vs 0%;100% vs 40%), PI3K was enriched in the SD/PD cohort.

**Figure 5:**
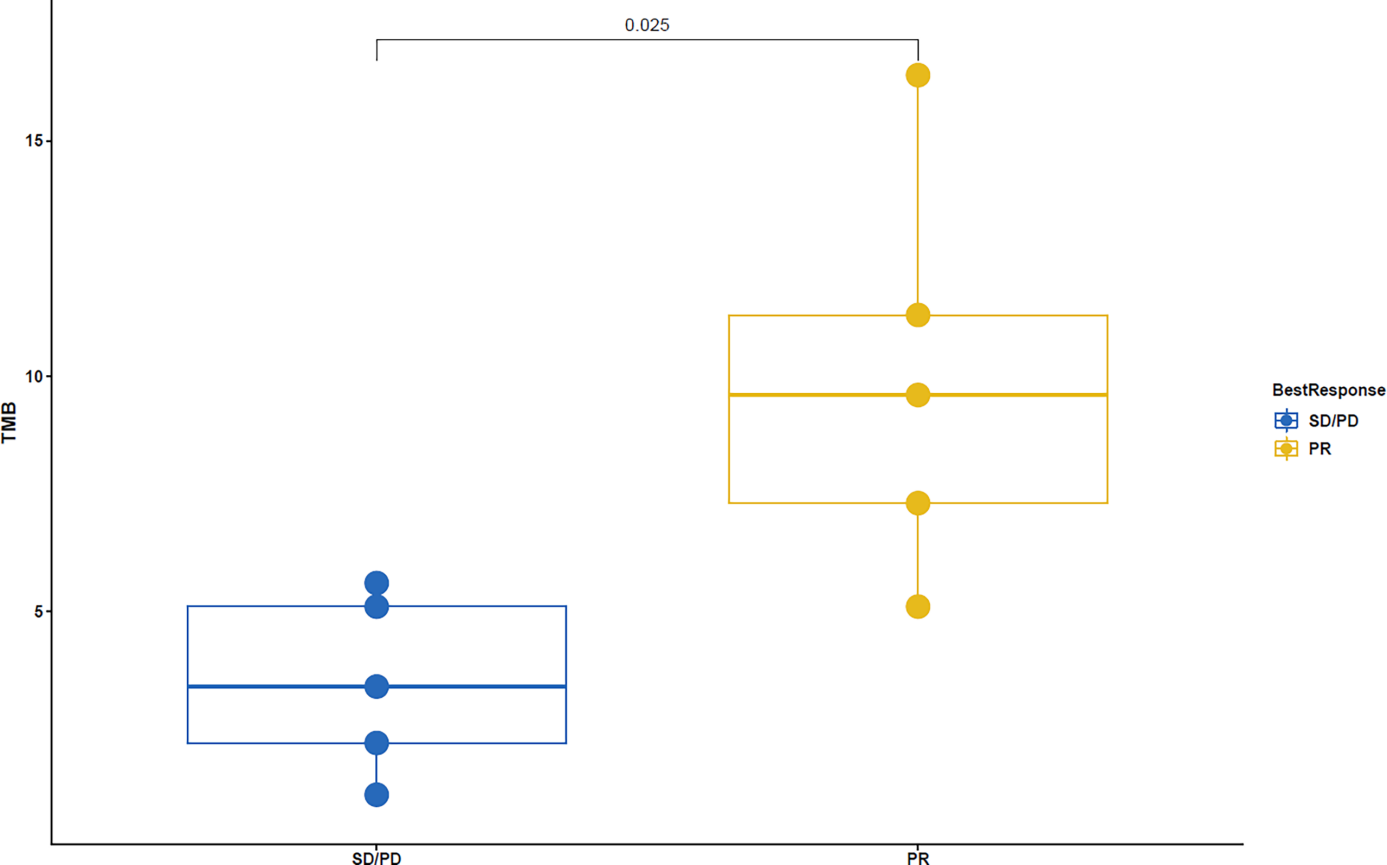
TMB showed significant statistical difference between PR and SD/PD (P=0.025).

The TMB of patients was detected, and there was a significant statistical difference between the two cohorts. TMB of the PR was significantly higher than that of the SD/PD (P=0.025) (**Figure 5**). In addition, in a patient with hyperprogression within two months of the combination treatment, multiple gene amplification on the chromosomes of 11q13-CCND1, FGF3, FGF4, and FGF19 were found in gene sequencing.

## Discussion

In terms of the current research, although immuno-targeted combination therapy has brought new hope to patients with advanced HCC, molecular biomarkers that can be applied to predict the efficacy of combination therapy are rarely systematically described due to the heterogeneity of HCC.

In this study, we preliminarily proved NLR through hematological sample analysis, CD3^+^CD4^+^CD279^+^ T lymphocytes, CD3^+^CD4^+^CD28^+^ T lymphocytes, and CD3^+^CD8^+^CD45RO^+^CD62L^+^T lymphocytes through lymphocyte subtype determination, and TP53, ARID1A, TMB, and 11q13 through genetic testing. These may be the predictive molecular biomarkers for screening effective populations for immuno-targeted combination therapy of HCC.

NLR is an inflammatory marker, which has been studied as a predictor of the efficacy of combination therapy for HCC. The median cut-off value of NLR is 4 (15). It has been confirmed that the increase in NLR has the same effect on OS in different tumor types, different sites, and different disease stages (HR=1.81, 95%CI=1.67-1.97; P <0.001), all suggesting a poor prognosis (15). Although there is no significant statistical difference in NLR in this research, there is still a trend that NLR in PR is lower compared with SD/PD. The sample size of this research is small, and the correlation between the NLR and HCC immuno-targeted combination therapy predictors still needs to be confirmed by a larger sample.

At present, only some studies believe that the detection of CD279^+^ (PD-1) T lymphocytes can better predict the clinical outcome of patients (16). Studies have also found that after treatment with PD-1 antibody for NSCLC, low PD-1 T lymphocytes indicate a poor prognosis (17). In addition, T cells with memory phenotypes (CD45RO^+^ and CD62L^+^) show better anti-tumor ability and better endurance both *in vivo* and *in vitro*(18), and related adoptive cell-transfer (ACT) therapy is also in the fiery research stage. In this study, the high expression of CD3^+^CD4^+^CD279^+^ and CD3^+^CD8^+^CD45RO^+^CD62L^+^ T lymphocytes reflects a correlation with a good prognosis. Nevertheless, whether CD279^+^, CD45RO^+^, and CD62L^+^ can be used as predictors of HCC combination therapy still needs further retrospective research.

CD28 is a co-stimulatory molecule expressed on the surface of activated T cells. It can promote the proliferation and differentiation T cells by binding to B7 molecules on antigen-presenting cells (APCs). Recent studies have pointed out that the efficacy of PD-1 antibody treatment is related to the proliferation of cytotoxic T lymphocytes (CTLs), and the proliferation of CTLs depends on CD28 co-stimulation (19). This finding indicates that the CD28 pathway may reverse the immuno-suppressive state. Furthermore, in lung adenocarcinoma, patients with high CD28 expression have lower disease-free survival (DFS) (20). The high expression of CD28 in the SD in our study was consistent with the above-mentioned conclusion, indicating that the high baseline status of CD28 might exhaust the ability of the co-stimulatory pathway to reverse immunosuppression, which led to the occurrence and development of tumors.

TP53 mutation is not only related to HCC staging, but also related to lower OS and recurrence-free survival (RFS) of patients (21). At present, studies have confirmed that lung cancer patients carrying TP53 or KRAS mutations have significant clinical efficacy on PD-1 antibody therapy, which can be used as a potential predictor of immunotherapy (22). In addition, ARID1A can exert a tumor suppressor effect by regulating the function of switching defective/sucrose non-fermenting (SWI/SNF) complex (23). At present, it is generally believed that the low expression of ARID1A is related to the poor prognosis of HCC (24), and patients with ARID1A mutations often get longer OS after immunotherapy (25). In this study, TP53 and ARID1A were enriched in the SD/PD cohort, and the contradictory conclusion might be attributed to the small test sample.

At present, many studies have confirmed that high TMB is related to the increased survival rate after immunotherapy for multiple tumor types. However, there is no uniform statement about the specific quantification of high TMB for different tumor types (26). The high TMB that appeared in the PR in this study was consistent with the above-mentioned statement, suggesting that it was a predictive factor for the efficacy of combination therapy for HCC.

Hyperprogression (HP) is closely related to the shortening of OS and PFS. At present, studies have found that the MDM2/MDM4 and copy number changes of several genes located on 11q13 are related to the HP of patients after treatment with ICIs (27). The 11q13 amplification mutation in hyperprogressive patients in this study was consistent with the above-mentioned conclusion, which preliminarily indicated that the immunotherapy was not effective for patients with 11q13 amplification mutation.

The above-mentioned laboratory indicators, lymphocyte subtypes, and gene mutations models might provide evidence for screening potentially beneficial populations for advanced HCC, although the number of patients in our study was not large enough. From our primary study, it was indicated that NLR, CD3^+^CD4^+^CD279^+^, CD3^+^CD4^+^CD28^+^, and CD3^+^CD8^+^CD45RO^+^CD62L^+^ T lymphocytes in peripheral blood, and the mutations of TP53, ARID1A, TMB, and 11q13 could predict the efficacy of immuno-targeted combination therapy for patients with advanced HCC. We will expand the number of samples and conduct a more in-depth exploration in future studies.

## Data Availability Statement

The original contributions presented in the study are included in the article/**Supplementary Material**. Further inquiries can be directed to the corresponding authors.

## Ethics Statement

The studies involving human participants were reviewed and approved by ethics committee of Comprehensive Cancer Center of Drum Tower Hospital of Nanjing University. The patients/participants provided their written informed consent to participate in this study.

## Author Contributions

JSo and BL conceived and designed the experiments. CL, SZ, YD, and JSn performed the experiments and analyzed the samples. CL, SZ, and YD analyzed the data. CL, SZ and YD composed the manuscript and provided figures. All authors interpreted the data, critically revised the manuscript for important intellectual contents and approved the final version.

## Funding

This study was supported by National Natural Science Foundation of China (No. 81902914); Jiangsu Provincial Medical Youth Talent (No. QNRC2016043); and the Key Medical Science and Technology Development Project of Nanjing (No. ZKX16032).

## Conflict of Interest

The authors declare that the research was conducted in the absence of any commercial or financial relationships that could be construed as a potential conflict of interest.

The reviewer SW declared a shared parent affiliation with the authors to the handling editor at the time of the review.

## Publisher’s Note

All claims expressed in this article are solely those of the authors and do not necessarily represent those of their affiliated organizations, or those of the publisher, the editors and the reviewers. Any product that may be evaluated in this article, or claim that may be made by its manufacturer, is not guaranteed or endorsed by the publisher.

## References

[1] Siegel RL Miller KD Jemal A. Cancer Statistics, 2018. CA Cancer J Clin (2018) 68(1):7–30. doi: 10.3322/caac.21442 29313949

[2] Lee MS Ryoo BY Hsu CH Numata K Stein S Verret W. Atezolizumab With or Without Bevacizumab in Unresectable Hepatocellular Carcinoma (GO30140): An Open-Label, Multicentre, Phase 1b Study. Lancet Oncol (2020) 21(6):808–20. doi: 10.1016/S1470-2045(20)30156-X 32502443

[3] Finn RS Qin S Ikeda M Galle PR Ducreux M Kim TY. Atezolizumab Plus Bevacizumab in Unresectable Hepatocellular Carcinoma. N Engl J Med (2020) 382(20):1894–905. doi: 10.1056/NEJMoa1915745 32402160

[4] Cheng A Qin S Ikeda M Galle PR Ducreux M Kim T. Updated Efficacy and Safety Data From IMbrave150: Atezolizumab Plus Bevacizumab vs. Sorafenib for Unresectable Hepatocellular Carcinoma. J Hepatol (2022) 76:862–73. doi: 10.1016/j.jhep.2021.11.030 34902530

[5] Finn RS Qin SK Ikeda M Galle PR Ducreux M Kim TY. IMbrave150: Updated Overall Survival (OS) Data From a Global, Randomized, Open-Label Phase III Study of Atezolizumab (Atezo) Plus Bevacizumab (Bev) Versus Sorafenib (Sor) in Patients (Pts) With Unresectable Hepatocellular Carcinoma (HCC). J Clin Oncol (2021) 39(3). doi: 10.1200/JCO.2021.39.3_suppl.267

[6] Finn RS Ikeda M Zhu AX Sung MW Baron AD Kudo M. Phase Ib Study of Lenvatinib Plus Pembrolizumab in Patients With Unresectable Hepatocellular Carcinoma. J Clin Oncol (2020) 38(26):2960–70. doi: 10.1200/JCO.20.00808 PMC7479760 32716739

[7] Latchman Y Wood CR Chernova T Chaudhary D Borde M Chernova I. PD-L2 is a Second Ligand for PD-1 and Inhibits T Cell Activation. Nat Immunol (2001) 2(3):261–8. doi: 10.1038/85330 11224527

[8] Freeman GJ Long AJ Iwai Y Bourque K Chernova T Nishimura H. Engagement of the PD-1 Immunoinhibitory Receptor by a Novel B7 Family Member Leads to Negative Regulation of Lymphocyte Activation. J Exp Med (2000) 192(7):1027–34. doi: 10.1084/jem.192.7.1027 PMC2193311 11015443

[9] Okazaki T Honjo T. PD-1 and PD-1 Ligands: From Discovery to Clinical Application. Int Immunol (2007) 19:813–24. doi: 10.1093/intimm/dxm057 17606980

[10] Llovet JM Ricci S Mazzaferro V Hilgard P Gane E Blanc JF. Sorafenib in Advanced Hepatocellular Carcinoma. N Engl J Med (2008) 359(4):378–90. doi: 10.1056/NEJMoa0708857 18650514

[11] Kudo M Finn RS Qin S Han KH Ikeda K Piscaglia F. Lenvatinib Versus Sorafenib in First-Line Treatment of Patients With Unresectable Hepatocellular Carcinoma: A Randomised Phase 3 non-Inferiority Trial. LANCET (2018) 391(10126):1163–73. doi: 10.1016/S0140-6736(18)30207-1 29433850

[12] Yau T Park JW Finn RS Cheng AL Mathurin P Edeline J. Nivolumab Versus Sorafenib in Advanced Hepatocellular Carcinoma (CheckMate 459): A Randomised, Multicentre, Open-Label, Phase 3 Trial. Lancet Oncol (2022) 23:77–90. doi: 10.1016/S1470-2045(21)00604-5 34914889

[13] Xu J Shen J Gu S Zhang Y Wu L Wu J. Camrelizumab in Combination With Apatinib in Patients With Advanced Hepatocellular Carcinoma (RESCUE): A Nonrandomized, Open-Label, Phase II Trial. Clin Cancer Res (2021) 27(4):1003–11. doi: 10.1158/1078-0432.CCR-20-2571 33087333

[14] Ren ZG Xu JM Bai YX Xu AB Cang SD Du CY. Sintilimab Plus a Bevacizumab Biosimilar (IBI305) Versus Sorafenib in Unresectable Hepatocellular Carcinoma (ORIENT-32): A Randomised, Open-Label, Phase 2-3 Study. Lancet Oncol (2021) 22(7):977–90. doi: 10.1016/S1470-2045(21)00252-7 34143971

[15] Templeton AJ McNamara MG Seruga B Vera-Badillo FE Aneja P Ocana A. Prognostic Role Of neutrophil-to-Lymphocyte Ratio in Solid Tumors: A Systematic Review and Meta-Analysis. J Natl Cancer Inst (2014) 106(6):u124. doi: 10.1093/jnci/dju124 24875653

[16] Kansy BA Concha-Benavente F Srivastava RM Jie H Shayan G Lei Y. PD-1 Status in CD8+T Cells Associates With Survival and Anti-PD-1 Therapeutic Outcomes in Head and Neck Cancer. Cancer Res (2017) 77(22):6353–64. doi: 10.1158/0008-5472.CAN-16-3167 PMC5690836 28904066

[17] Kamphorst AO Pillai RN Yang S Nasti TH Akondy RS Wieland A. Proliferation of PD-1+ CD8 T Cells in Peripheral Blood After PD-1–Targeted Therapy in Lung Cancer Patients. Proceedings of the National Academy of Sciences (2017) 114(19):4993–8. doi: 10.1073/pnas.1705327114 PMC5441721 28446615

[18] Liu Q Sun Z Chen L. Memory T Cells: Strategies for Optimizing Tumor Immunotherapy. Protein Cell (2020) 11(8):549–64. doi: 10.1007/s13238-020-00707-9 PMC7381543 32221812

[19] Kamphorst AO Wieland A Nasti T Yang S Zhang R Barber DL. Rescue of Exhausted CD8 T Cells by PD-1–Targeted Therapies Is CD28-Dependent. Science (2017) 355(6332):1423–7. doi: 10.1126/science.aaf0683 PMC5595217 28280249

[20] Sun D Tian L Bian T Zhao H Tao J Feng L. CD28 in the Prognosis of Young Lung adenocarcinoma Patients. Bmc Cancer (2020) 20(1):910. doi: 10.1186/s12885-020-07412-0 32967633 PMC7510131

[21] Liu J Ma Q Zhang M Wang X Zhang D Li W. Alterations of TP53 Are Associated With a Poor Outcome for Patients With Hepatocellular Carcinoma: Evidence From a Systematic Review and Meta-Analysis. Eur J Cancer (2012) 48(15):2328–38. doi: 10.1016/j.ejca.2012.03.001 PMC3395767 22459764

[22] Dong ZY Zhong WZ Zhang XC Su J Xie Z Liu SY. Potential Predictive Value of TP53 and KRAS Mutation Status for Response to PD-1 Blockade Immunotherapy in Lung Adenocarcinoma. Clin Cancer Res (2017) 23(12):3012–24. doi: 10.1158/1078-0432.CCR-16-2554 28039262

[23] Kadoch C Hargreaves DC Hodges C Elias L Ho L Ranish J. Proteomic and Bioinformatic Analysis of Mammalian SWI/SNF Complexes Identifies Extensive Roles in Human Malignancy. Nat Genet (2013) 45(6):592–601. doi: 10.1038/ng.2628 23644491 PMC3667980

[24] Yim S Kang S Shin J Jeong Y Sohn B Um S. Low ARID1A Expression is Associated With Poor Prognosis in Hepatocellular Carcinoma. CELLS-BASEL (2020) 9(9):2002. doi: 10.3390/cells9092002 PMC7564185 32878261

[25] Jiang T Chen X Su C Ren S Zhou C. Pan-Cancer Analysis Ofarid1a Alterations as Biomarkers for Immunotherapy Outcomes. J Cancer (2020) 11(4):776–80. doi: 10.7150/jca.41296 PMC6959029 31949479

[26] Samstein RM Lee C Shoushtari AN Hellmann MD Shen R Janjigian YY. Tumor Mutational Load Predicts Survival After Immunotherapy Across Multiple Cancer Types. Nat Genet (2019) 51(2):202–6. doi: 10.1038/s41588-018-0312-8 PMC6365097 30643254

[27] Singavi AK Menon S Kilari D Alqwasmi A Ritch PS Thomas JP. 1140pdpredictive Biomarkers for Hyper-Progression (HP) in Response to Immune Checkpoint Inhibitors (ICI) – Analysis of Somatic Alterations (SAs). Ann Oncol (2017) 28. doi: 10.1093/annonc/mdx376.006

