# Supplementary material for: The Potential Predictive Biomarkers for Advanced Hepatocellular Carcinoma Treated With Anti-Angiogenic Drugs in Combination With PD-1 Antibody
Source: Front Immunol. 2022 Jul 7;13:930096. doi: 10.3389/fimmu.2022.930096 (PMC9301374; doi:10.3389/fimmu.2022.930096)
Supplement: Supplementary file 1 [file DataSheet_1.docx]

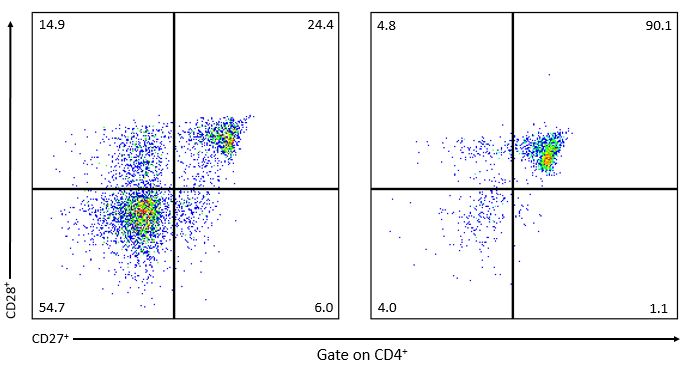


Patient 10

Patient1


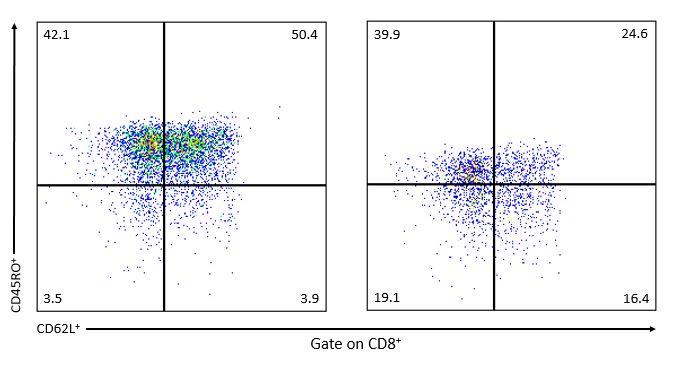

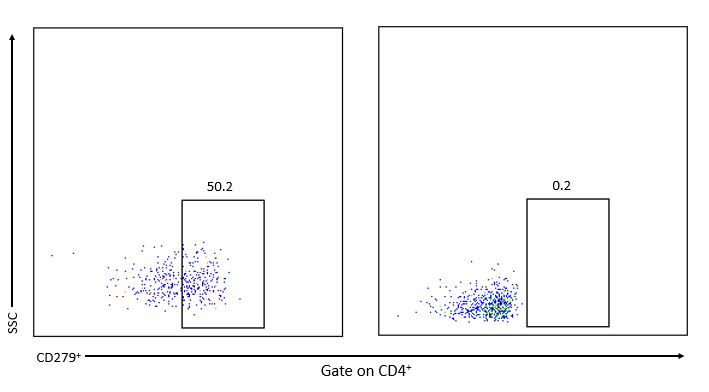


C

**sFig 1** Different T-cell subsets in peripheral blood mononuclear cells (PBMCs) of the patient 1 (PR) and patient 10 (SD). **A**: CD3^+^CD4^+^CD28^+^ T-cell subsets, **B**: CD3^+^CD4^+^CD279^+^ T-cell subsets, **C**: CD3^+^CD8^+^CD45RO^+^CD62L^+^ T-cell subsets

B

A
